# Tobacco Use, Food Insecurity, and Low BMI in India’s Older Population

**DOI:** 10.3390/nu16213649

**Published:** 2024-10-26

**Authors:** Yesuvadian Selvamani, Jalandhar Pradhan, Joelle H. Fong

**Affiliations:** 1 School of Public Health, SRM Institute of Science and Technology, Kattankulathur, Chennai 603203, India; selvamay@srmist.edu.in; 2 Department of Humanities and Social Sciences, National Institute of Technology, Rourkela 769008, India; 3 Lee Kuan Yew School of Public Policy, National University of Singapore, 469C Bukit Timah Road, Singapore 259771, Singapore

**Keywords:** smoking, food insecure, malnutrition, population aging, body mass index

## Abstract

Background: Underweight is a prevalent condition among older adults in developing countries and poses a substantial burden on health, social, and aged-care systems. In this study, we examined the influence of tobacco use and food insecurity on the risk of being underweight among adults aged 60 or older in India. Methods: We used data from the 2017–2018 Longitudinal Aging Study in India. The sample size was 27,902 adults aged 60 years and above. We distinguished between smoking and smokeless tobacco use to examine how they may independently contribute to the outcome, while food insecurity was measured using the four-item version of the Food Insecurity Experience scale. Multivariable logistic regressions were conducted to assess the impact of tobacco use and food insecurity on the outcome. Additionally, we tested interactions between tobacco use and education, as well as between tobacco use and wealth. Results: The prevalence of underweight was 27% in the study population. Older adults who used smoking tobacco were twice more likely to be underweight than non-users (OR = 2.07, 95% CI = 1.79 to 2.40, *p* < 0.001), controlling for other confounders. The positive association between smokeless tobacco use and underweight was weaker but still significant (OR = 1.26, 95% CI = 1.11 to 1.42, *p* < 0.001). Food-insecure older adults were also more likely to be underweight (OR = 1.27, 95% CI = 1.10 to 1.48, *p* < 0.001). Other risk factors of underweight included males, rural residents, lower socioeconomic status (in terms of education, wealth, and caste), chewing disability, poor self-rated health, chronic lung disease, and tuberculosis. Interestingly, tobacco use moderated the relationship between wealth and underweight, such that smokers are more likely to be underweight as they become more affluent. Conclusions: Tobacco use and food insecurity have adverse implications on the nutritional status of the older persons in developing countries such as India. More targeted tobacco cessation measures and active food assistance programs for older adults are warranted to improve the overall health status of the older demographic.

## 1. Introduction

Underweight and its associated health effects among adults are quickly becoming serious health concerns in low- and middle-income countries. India is no exception, where about 10–14% of adult men and women have low body mass index (BMI), with higher prevalence rates documented among adolescents and women [1,2]. Underweight in adulthood, defined as BMI < 18.5 kg/m^2^, can have significant adverse health effects and has been found to be associated with non-communicable illnesses and functional limitations [3], higher mortality risk [4], infertility and adverse perinatal outcomes [5], as well as lower self-rated health and quality of life [6]. Past research has identified a variety of risk factors that contribute to adult underweight. These include sociodemographic factors, such as age, lower education, working for pay, lower socioeconomic status, and residing in rural areas [1,7,8]. Health-related risk factors for adult underweight include poor diets with inadequate nutrient density, tobacco use, frailty, dementia, eating disorders, and other health conditions that may cause regular nausea or vomiting [2,9,10,11].

In this study, we explore the impact of tobacco use and food insecurity on the risk of being underweight among adults aged 60 or older in India. Underweight (a form of malnutrition) is a highly prevalent condition in older adults in India and poses a substantial burden on health, social, and aged-care systems. Notably, a nationwide household survey that covered all 30 states and the six Union Territories of India found that more than a quarter of adults aged 60 or older are underweight. In addition, the prevalence of underweight among the elderly is almost threefold higher in rural areas than in urban areas [8]. At the same time, India is rapidly aging due to projected declines in the total fertility rate and increases in life expectancy. By 2050, the share of older adults aged 60 and above is expected to be 20.8% [12]. It is, thus, critical to examine the risk factors contributing to low BMI in the older adult population in India.

Our study builds on a few prior papers, which have evidenced a strong positive association between tobacco use and low BMI among adolescents [13], as well as the general adult population [14,15,16]. For instance, using a sample of American adults aged 25–64, Plurphanswat et al. [15] found that both men and women current smokers were more likely to be underweight and normal weight compared to never smokers. Wang et al. [13] focused on the link between underweight and tobacco use among adolescents in 23 low-income and middle-income countries. Pednekar et al. [14] showed that both smoking and smokeless tobacco are positively associated with low BMI among individuals aged 35 years and older in the city of Mumbai. Less is known, however, about how tobacco use affects the weight of older adults. In addition, it is unclear whether and how different forms of tobacco use (smoking versus smokeless tobacco) are associated with low BMI in the older adult population. This study attempts to close these research gaps.

Tobacco has traditionally been used in various forms in India and other Asian countries. While tobacco smoking involves the burning of tobacco products inhaled through the mouth, smokeless tobacco is consumed without burning the product and can be used orally or nasally. The more prevalent form of tobacco use in India is smokeless tobacco, and such products include khaini, gutkha, betel quid with tobacco and zarda [17]. Zarda, for instance, consists of tobacco, lime, spices and vegetable dyes. The smoking forms of tobacco used are cigarettes, bidi, and hookah. Bidis are handmade cigarettes obtained by rolling a dried rectangular piece of temburni leaf with tobacco. According to the World Health Organization, India is the second leading tobacco consumer in the world, with over 260 million adults, or about 29% of all adults, using tobacco [18]. More than 30% of the middle-aged and older adults use some form of tobacco [8]. Accordingly, in this present study, we distinguish between smoking and smokeless tobacco use and examine how they may independently contribute to the health condition of being underweight among older adults in India.

Past research has also highlighted that a lack of food security may increase the risk of malnutrition in all of its forms, including being underweight [19,20,21]. Groups of the population that are more vulnerable to food insecurity, and, thus, more susceptible to dietary inadequacies, include pregnant mothers, families with children, the elderly, and people on a low income. Indeed, Fong [22] estimates that the prevalence of food insecurity—the lack of regular access to enough safe and nutritious food for an active and healthy life—was about 10.5% among older individuals aged 60 and above in India. Older Indian adults who were male, younger, less educated, socially disadvantaged, in rural areas, and outside the northern region were most vulnerable to food insecurity [22]. Another study found that both food insecurity and underweight are widespread among older adults residing in central, eastern, and north-eastern India; those who are food insecure are significantly more likely to be underweight [20]. Nonetheless, that study did not account for older adults’ tobacco use, which is potentially a key risk factor for being underweight. Our analysis in this present study is, thus, more comprehensive in accounting for both the possible impact of tobacco use and food insecurity on the risk of being underweight among older adults, controlling for other standard socio-demographic personal characteristics.

## 2. Materials and Methods

### 2.1. Study Design and Sample

**Figure 1:**
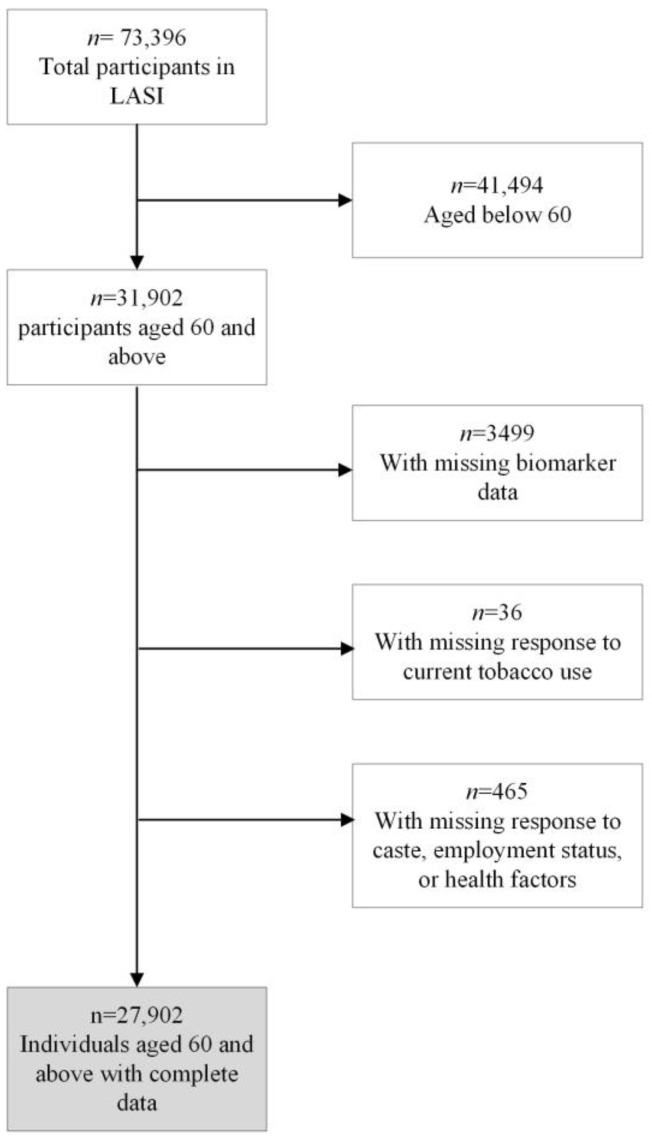
Flowchart of the study sample, LASI, 2017–2018.

This study uses secondary data from the first wave of the Longitudinal Ageing Study in India (LASI), 2017-18. LASI is India’s first and the world’s largest study that provides a longitudinal database for the middle-aged and older population in the wide-ranging domains of social, health, and economic wellbeing. The LASI survey collected data for older adults aged 45 and above and their spouse, irrespective of age. The survey also provides representative data for all states and Union Territories of India to understand the sub-national variations in social, health, and economic wellbeing outcomes. The overall sample of the LASI study is 73,396. After excluding subjects younger than 60 years, as well as those with no biomarker data or missing key variables, our final analytical sample is 27,902 (see Figure 1). LASI adopts a multi-stage sampling method and provides rich information on socio-demographic characteristics, biomarkers based on health assessment, dietary patterns, tobacco use, health conditions, self-reported health, and so on. The LASI study was approved by the Indian Council of Medical Research Ethics Committee and written/oral informed consent was obtained from the participants; detailed information about LASI is presented elsewhere [8].

### 2.2. Measures

#### 2.2.1. Dependent Variable

Body mass index is an important indicator used to assess an older adult’s nutritional status [8]. In the LASI survey, the height and weight of older adults were measured using standard procedures. We computed the BMI of an individual as the ratio of weight (in kilograms) to the square of height (in meters). For this study, the WHO classification was adopted to classify the BMI in terms of underweight, normal weight, and overweight: underweight if BMI < 18.5 kg/m^2^; normal weight if BMI in the range of 18.5–24.9 kg/m^2^; overweight/obese if BMI ≥ 25 kg/m^2^. A binary dependent variable was constructed and set to 1 if underweight and 0 otherwise.

#### 2.2.2. Key Explanatory Variables

Tobacco use. In the LASI survey, first, respondents were asked about tobacco use through this question: “Have you ever smoked tobacco (cigarette, bidi, cigar, hookah, cheroot) or used smokeless tobacco (such as chewing tobacco, gutka, pan masala, etc.)?” Those who responded yes were further asked about what types of tobacco product they have used or consumed. Answer options included “smoke tobacco”, “smokeless tobacco”, and “both smoke and smokeless tobacco”. Using responses to both questions, we constructed a categorical variable for tobacco use, which comprised four categories as follows: (1) currently not using any form of tobacco, (2) using smoke tobacco, (3) using smokeless tobacco, and (4) using both forms of tobacco.

Food insecurity. Following Fong [22] and other studies [21], food insecurity among older adults was assessed using four items in the LASI survey. The food insecurity variable is an indicator variable set to 1 if the respondent provided an affirmative response to any of the four questions (listed below) and 0 otherwise.

1. In the last 12 months, did you reduce the size of your meals or skip meals because there was not enough food at your household?
2. In the last 12 months, were you hungry but did not eat because there was not enough food at your household?
3. In the past 12 months, did you ever not eat for a whole day because there was not enough food at your household?
4. Do you think that you have lost weight in the last 12 months because there was not enough food in your household?

#### 2.2.3. Other Controls

We considered a host of other potential covariates, including demographic factors (age, sex, marital status, place of residence, and region), socio-economic factors (education, work status, caste, religion, and household economic status), as well as health factors (chewing difficulty, self-rated health, functional limitations, acute diseases, and chronic diseases). 

Demographic factors. Three age group categories were defined (60–69, 70–79, and ≥80 years). Separate binary variables were used for female (reference group is male) and urban place of residence (reference group is rural). Marital status in LASI was assessed with several categories as follows: 1. currently married, 2. widowed, 3. divorced, 4. separated, 5. deserted, 6. live-in relationship, 7. never married. In our analysis, we recoded 1 as currently married and 2–7 as others. Since India is a large country, we captured cross-state heterogeneity using a region variable (North, Central, East, Northeast, West and South).

Socio-economic factors. The educational attainment of the study participants was categorized as 1. no schooling, 2. middle or less, and 3. at least secondary. A binary variable was used for work status and set to 1 if currently working and 0 otherwise. In India, the caste system measures social stratification, which is similar to race/ethnicity in the global context. In this study, caste was categorized as 1. Scheduled Tribe (ST), 2. Scheduled Caste (SC), 3. Other Backward Class (OBC) and 4. Others. Caste-based inequality in child and adult health, nutrition, and socio-economic development is strongly evident in India, with those in certain caste groups such as ST and SC tending to be more disadvantaged [23,24]. Religion was categorized as 1. Hindu, 2. Muslim, 3. Christian, and 4. Others (None, Sikh, Buddhist/Neo-Buddhist, Jain, Jewish, Parsi/Zoroastrian and other). Previous studies also show a close association between economic status and underweight [6]. To measure the general economic status and standard of living of the household, we used monthly per capita consumption expenditure (MPCE) quintiles from the LASI survey.

Health factors. Chewing ability in old age is associated with poor diet intake, nutrition, and risk for healthy ageing [25,26]. In the LASI survey, chewing disability was assessed using the following question “How well can you chew solid foods such as chapati, apple, guava, or nuts?” Subjects who said not well and not at all were classified as having chewing difficulty, whereas those who gave other responses (very well, pretty well, or fairly well) were without such difficulty. We generated an indicator variable for poor self-rated health set to 1 if the respondent answered poor or very poor to the question of “Overall, how is your health in general?” Functional limitations of older adults were based on activities of daily living (ADL) and instrumental activities of daily living (IADL) measures. The indicator variable for 1+ ADL limitations was set to 1 if the respondent had difficulty dressing, walking across a room, bathing, eating, getting in or out of bed, or using the toilet and 0 otherwise. Similarly, the 1+ IADL limitations were set to 1 if the respondent had difficulty with any of the seven IADL items and 0 otherwise. The IADL items assessed in the LASI survey are: (1) preparing meal, (2) shopping for groceries, (3) making telephone calls, (4) taking medications, (5) doing work around the house or garden, (6) managing money, and (7) getting around or finding addresses in an unfamiliar place. To account for the presence of acute diseases, which are fairly common in India, even among older adults, we included in the analysis separate binary variables for jaundice/hepatitis, tuberculosis, malaria, diarrhoea/gastroenteritis, and anemia. Finally, we included seven separate binary variables for hypertension, stroke, chronic heart diseases, cancer, chronic lung disease, diabetes, and arthritis.

### 2.3. Statistical Analysis

Bivariate and multivariate analyses were implemented on the weighted sample. Bivariate analyses provided an understanding of the sample profile, as well as how the prevalence of being underweight differed by tobacco use, food insecurity, and other demographic and socioeconomic characteristics. Chi-squared tests were performed. Further, we conducted multivariate logistic regression to obtain the adjusted odds of tobacco use and food insecurity on underweight. We used five different regression models: model 1 with all study participants aged 60 and above, while models 2 and 3 stratify the sample by gender (male versus female). In models 4 and 5, the sample was stratified by place of residence (rural versus urban dwellers). Finally, to draw deeper insights, we also tested interactions between tobacco use and education attainment, as well as between tobacco use and MPCE wealth quintile. Regression results are presented in odds ratio (OR) with 95% confidence interval. Individual-level weights were used in all the analyses to ensure the results are generalizable to the older population. All analysis was carried out using STATA 18.

## 3. Results

### 3.1. Sample Attributes and Prevalence of Underweight by Socio-Demographic Characteristics

Table 1 presents the characteristics of the study population (*N* = 27,902). In the weighted sample, the prevalence of being underweight was 27%. About 20% of the older adults used smokeless tobacco only, 12% used smoking tobacco only, while 3% used both forms of tobacco. The prevalence of food insecurity was 11%. The bulk of the study participants (60%) belonged to the age group of 60–69. The share of women participants was 52%. Almost three-quarters of the respondents (72%) resided in rural areas, and 45% belonged to the Other Backward Class (OBC) caste group. The share of older adults identifying as Hindu was the highest at 82%, followed by Muslims (11%), then Christians (2.8%). The prevalence of chewing disability was 33%. More than half of the respondents had no schooling, while 32.2% were currently working. In the weighted sample, about a quarter of the participants had poor self-rated health, 22% had 1+ ADL limitations, and 47% had 1+ IADL limitations. More than 30% of the study participants reported that they have been diagnosed with hypertension. The prevalence of diabetes was 14%, and nearly one in five reported arthritis.

Our bivariate analysis shows that the prevalence of being underweight differed by tobacco use, food insecurity, and other personal characteristics. Specifically, the prevalence of being underweight was higher among those who used tobacco (either smoking, smokeless or both forms) as compared to those who did not use any tobacco. The prevalence of underweight was also higher among older adults with food insecurity, as compared to their counterparts who were food secure. The *p*-values of the respective chi-squared tests were statistically significant at the *p* < 0.001 level. We also observe that the prevalence of underweight was notably higher among males, those of more advanced ages, those with chewing disabilities, as well as among those residing in rural areas. In contrast, the prevalence of underweight decreased along education and wealth gradients. Larger proportions of older Indian adults in the ST and SC caste groups were underweight as compared to their counterparts in other caste categories.

### 3.2. Multivariate Logistic Regression Results

Table 2 presents the multivariate logistic regression results for the full sample and the stratified samples. Focusing first on results for the overall sample (column 1), we find that the association of tobacco use on underweight is statistically significant and positive. Older adults who used smoking tobacco were two-times more likely to be underweight than non-users of tobacco (OR = 2.07, 95% CI = 1.79 to 2.40, *p* < 0.001). The positive association between smokeless tobacco use and underweight is weaker but still statistically significant (OR = 1.26, 95% CI = 1.11 to 1.42, *p* < 0.001). Older adults using both smoke and smokeless tobacco were at higher risk of being underweight than non-users (OR = 1.74, 95% CI = 1.36 to 2.24, *p* < 0.001). The association between food insecurity and underweight is statistically significant and positive (OR = 1.27, 95% CI = 1.10 to 1.48, *p* < 0.001).

Upon comparing adjusted odds ratios for tobacco use and food insecurity with unadjusted odds ratios (not reported here for brevity), the adjusted odds ratios were systematically smaller, indicating that the control variables used, including the health factors, were confounders. Females, urban residents, persons with higher educational attainment, and persons in higher wealth quintiles were less likely to be underweight. Respondents in the ST caste group were significantly more likely to be underweight as compared to other caste groups. Other factors positively correlated with being underweight included being unmarried, having chewing disability, having poor self-rated health, having one or more IADL limitations, having chronic lung disease, as well as acute diseases, such as tuberculosis, malaria, and anemia.

Our empirical estimates in columns (2) and (3) of Table 2 also reveal gender differences in terms of how tobacco use correlates with the outcome examined. Specifically, we find that the association between different forms of tobacco use and being underweight is stronger among older women than older men. The magnitudes of the ORs for the female subsample are larger: females who used smoking tobacco were 2.15 (*p* < 0.001) times more likely to be underweight (compare OR = 1.93, *p* < 0.001 for males). In fact, females who used both smokeless and smoking tobacco were 4.26 (*p* < 0.001) times more likely to be underweight (compare OR = 1.55, *p* < 0.001 for males). This is partly because the relationship between smokeless tobacco use and underweight is statistically significant for women (OR = 1.46, 95% CI = 1.24 to 1.73, *p* < 0.001) but not men. Sample stratification by place of residence shows that the association between different forms of tobacco use and underweight is stronger for rural residents than urban residents (see columns 4 and 5). The impact of food insecurity on being underweight remains statistically significant and positive across these various subsamples considered, except for the case of the female subsample in column (3).

Our findings, thus far, suggest that tobacco use plays a key role in explaining older adult underweight. To examine how socio-economic status may interact with tobacco use to affect the outcome, we performed additional analysis by including interaction terms for tobacco use and education and, separately, for tobacco use and the wealth quintile. Regression results with interaction terms are presented in Table 3. The association between education and being underweight did not substantively differ by tobacco use (model 1). By contrast, the association between wealth level and being underweight differed by tobacco use in India (model 2 in Table 3). As wealth increases for non-users of tobacco, they are less likely to be underweight. However, for users of tobacco, they are more likely to be underweight as they become more affluent. This is especially the case for those older adults using both smoking and smokeless tobacco (model 2, OR = 2.97, 95% CI = 1.55 to 5.70, *p* < 0.001).

## 4. Discussion

This study assessed the impact of tobacco use and food insecurity on the risk of being underweight among adults aged 60 or older in India using the LASI, 2017–2018, a nationally representative dataset. The findings suggested that both smoking and smokeless tobacco use were positively and significantly associated with being underweight. Furthermore, the positive relationship between smoking tobacco and underweight was stronger and more consistent across population subgroups, as compared to that of smokeless tobacco. Older Indian adults who smoked tobacco were twice as likely to be underweight compared to non-users of tobacco, particularly among women and among rural residents. The association between food insecurity and being underweight was also positive and statistically significant, underscoring the importance of food access as a determinant of the nutritional status of the older population. Socio-economic status measured by education and wealth generally demonstrated a negative association with underweight, which suggests that older adults with higher socioeconomic status were less likely to be underweight. Nonetheless, additional analysis with interaction terms revealed that the association between wealth and being underweight differed by tobacco use. Older people who used tobacco, of whatever form, were more likely to be underweight as they became more affluent, while the opposite holds true for non-users of tobacco.

Our results pertaining to the older demographic are consistent with past evidence that tobacco use is positively associated with low BMI among adolescents and the wider population at large [13,14,15,16]. The pathways of the impact of tobacco use on BMI are diverse. For instance, some studies highlight that nicotine increases energy expenditure and reduced appetite, which may explain why smokers tend to have lower body weight than non-smokers [27,28]. In other words, nicotine may suppress one’s appetite, resulting in reduced food intake. Over time, this may lead to lower body weight and, consequently, lower BMI among older adults whose height is likely fixed. The stronger association observed between smoking tobacco and underweight, as compared to smokeless tobacco and underweight, may be attributable to the different usage patterns for different forms of tobacco. Specifically, there is evidence that the daily frequency of smoking tobacco usage is higher on average than that for smokeless tobacco in India [29]. Also, the attempts to quit tobacco are lower among smoking tobacco users than the smokeless tobacco users [30].

The positive and statistically significant association between food insecurity and being underweight is consistent with the findings in a previous study, also using data from India [20]. Our further analysis using stratified samples confirmed that the impact of food insecurity on being underweight holds across various subsamples considered, except for the case of the female subsample, although the OR (which exceeded one) was in the right direction. Food insecurity plays an important role in determining the nutritional status of the population as it encompasses both the access to food and the quality of the diet. In this perspective, even older adults who have regular access to food may be exposed to the risk of food insecurity due to unsafe or non-nutritious food. In India, about 1 in 10 older adults are food insecure [22], with males, the younger old, the less educated, the socially disadvantaged, and rural residents being most vulnerable to food insecurity. Also, having limited access to nutritious food may lead to the initiation and/or escalation of tobacco use to help alleviate psychological stress [31,32], which is another potential pathway in which food insecurity may influence BMI status.

In terms of other risk factors, the results showed that older persons in India who were males, rural residents, not married, less wealthy, with lower educational attainment, in the ST caste group, have chewing disability, with poor self-rated health, have IADL limitations, have chronic lung disease, and have acute diseases (tuberculosis, malaria, and anemia) were significantly more likely to be underweight. The positive association between having chewing disability and being underweight is broadly consistent with findings in other studies [25,26], which underscores the importance of oral health in determining BMI and nutritional status. Our analysis highlighted that the prevalence of underweight is systematically lower among older adults with lower socio-economic status, irrespective of the socio-economic status measure used (education, caste, or wealth). Older Indian adults in the ST caste group, lowest wealth quintile, and with no schooling face a high risk of being underweight. Caste-based differences in adult nutritional status and BMI have also been reported in previous studies [7,33,34,35].

An interesting finding in the context of India is that the association between tobacco use and underweight was stronger for older adults residing in rural areas. This may be due to the higher burden of tobacco use in rural areas along with other vulnerabilities such as second-hand smoke and higher levels of indoor air pollution (that may arise due to the use of dirty energy or other causes). A multi-country study conducted in 14 low- and middle-income countries suggests a significant association between indoor air pollution and underweight among children and adult populations [36]. Another notable finding from our study is positive association between tuberculosis and underweight in India, which is in line with previous studies, highlighting a higher risk of mortality attributable to tuberculosis among persons who are underweight [37]. We found that poor self-rated health was positively and strongly correlated to older adult underweight, not only in the full sample but also across the various demographic subgroups considered. In contrast, the correlation between functional disabilities (ADL and IADL limitations) and underweight was much weaker in the older Indian adult population.

The strength of this study is the use of community-based and nationally representative data from the older population. Also, to the best of our knowledge, this is the first study to examine the association between different forms of tobacco use and underweight among older adults in India using nationally representative data. The additional insight that tobacco use moderates the relationship between wealth and being underweight is also valuable in designing policy interventions to improve the nutritional status of the growing older population in India. This study has a few limitations. First, the data used in this study are cross-sectional. Therefore, the causal association link cannot be established. In this perspective, the use of longitudinal data to examine the association between the type of tobacco use and underweight will be important. Second, the measurement of tobacco use is based on self-reports. It is possible for survey participants, especially women, to under-report their actual usage of tobacco due to the lack of social desirability of smoking, which is reportedly quite evident in the context of India [38].

## 5. Conclusions

Tobacco use, food insecurity, and malnutrition are all serious public health issues in India and other developing countries. Our findings suggest that tobacco use and food insecurity are both important risk factors of older adult underweight in India, controlling for other socio-demographic and health characteristics. Tobacco use, in particular, may have even more far-reaching public health implications in India than previously thought. Although usage of smokeless tobacco has a smaller negative impact on BMI than usage of smoking tobacco, its effect is still significant and nontrivial. India is the second leading tobacco consumer in the world. Targeted interventions such as tobacco cessation measures focusing on older adults residing in rural areas, women, and those with lower socio-economic status will be important. Such interventions can benefit other public health goals on improved nutritional status and consequential health benefits. At the same time, active food assistance programs catering to older persons are needed in order to establish a food-secure environment for the rapidly aging society of India. Most of the community-based programs currently target mainly children and women, including, for instance, the Mid-Day Meal scheme and the widespread network of the Anganwadi centers (which provide basic healthcare in Indian villages) rolled out by the Integrated Child Development Services. As societies continue to age, dedicated programs to address older adults’ tobacco use and food insecurity are urgently warranted.

## Figures and Tables

**Table 1:** Characteristics of the study population, Longitudinal Ageing Study in India wave 1, 2017–2018.

Variables	Full Sample		Subsamples Based on Underweight Status		*p*-Value
	Total (*N* = 27,902)	Number	No (*n* = 21,456)	Yes (*n* = 6446)	
Underweight					
No	73.1%	21,456			
Yes	27.0%	6446			
Tobacco use					
No	65.3%	18,962	81.6%	18.4%	
Smoking	12.3%	3428	63.1%	36.9%	
Smokeless	19.6%	4719	70.0%	30.0%	
Both	2.8%	793	65.3%	34.6%	<0.001
Food insecure					
No	89.4%	25,440	77.9%	22.6%	
Yes	10.6%	2462	66.1%	33.9%	<0.001
Age					
60–69	60.0%	17,140	79.9%	20.1%	
70–79	29.8%	8038	74.2%	25.8%	
80+	10.3%	2724	66.0%	34.0%	<0.001
Female					
No	47.7%	13,487	76.3%	23.7%	
Yes	52.3%	14,415	77.5%	22.6%	0.023
Urban residence					
No	72.4%	18,715	71.1%	28.9%	
Yes	27.6%	9187	88.7%	11.3%	<0.001
Currently married					
Yes	62.7%	17,891	78.9%	21.1%	
No	37.3%	10,011	73.3%	26.7%	<0.001
Region					
North	13.0%	5143	82.0%	18.0%	
Central	21.2%	3750	63.8%	36.2%	
East	24.6%	5233	68.3%	31.7%	
Northeast	3.0%	3659	77.6%	22.4%	
West	16.6%	3586	80.0%	20.0%	
South	21.5%	6531	85.2%	14.8%	<0.001
Education attainment					
No schooling	56.7%	14,961	70.3%	29.8%	
Middle or less	29.4%	8797	81.1%	18.9%	
At least secondary	13.9%	4144	92.0%	8.0%	<0.001
Currently working					
Yes	32.2%	8460	74.6%	25.4%	
No	67.8%	19,442	77.9%	22.1%	<0.001
Caste					
Scheduled Tribe	8.5%	4681	72.5%	27.5%	
Scheduled Caste	19.3%	4572	70.4%	29.6%	
Other backward class	45.1%	10,703	76.4%	23.6%	
None of above	27.1%	7946	83.9%	16.1%	<0.001
Religion					
Hindu	82.2%	20,345	74.9%	25.1%	
Muslim	11.3%	3262	80.9%	19.2%	
Christian	2.8%	2816	82.9%	17.1%	
Others	3.7%	1479	84.6%	15.4%	<0.001
Wealth quintiles (MPCE)					
Lowest	21.8%	5689	68.6%	31.4%	
Second	21.8%	5764	71.8%	28.2%	
Middle	20.9%	5738	77.4%	22.6%	
Fourth	19.1%	5495	81.4%	18.6%	
Highest	16.4%	5216	86.1%	13.9%	<0.001
Poor self-rated health					
No	76.3%	21,678	77.7%	22.3%	
Yes	23.7%	6224	74.0%	26.0%	<0.001
Chewing disability					
No	66.8%	18,995	80.5%	19.5%	
Yes	33.2%	8932	69.2%	30.8%	<0.001
1+ ADL					
No	77.9%	22,448	77.4%	22.6%	
Yes	22.1%	5454	74.9%	25.1%	<0.001
1+ IADL					
No	53.1%	15,938	79.7%	20.3%	
Yes	46.9%	11,964	73.1%	26.9%	<0.001
Hypertension					
No	67.8%	18,194	71.5%	28.5%	
Yes	32.2%	9708	87.1%	13.0%	<0.001
Stroke					
No	97.6%	27,277	76.7%	23.3%	
Yes	2.4%	625	85.4%	14.6%	<0.001
Chronic heart diseases					
No	94.7%	26,499	76.2%	23.8%	
Yes	5.29%	1403	89.6%	10.4%	<0.001
Cancer					
No	99.4%	27,703	76.9%	23.2%	
Yes	0.6%	199	82.9%	17.1%	0.043
Chronic lung disease					
No	91.6%	25,823	77.5%	22.5%	
Yes	8.36%	2079	69.6%	30.5%	<0.001
Diabetes					
No	86.1%	23,638	74.0%	26.0%	
Yes	13.9%	4264	92.9%	7.1%	<0.001
Arthritis					
No	80.8%	22,994	75.5%	24.5%	
Yes	19.2%	4908	83.3%	16.7%	<0.001
Jaundice/hepatitis					
No	97.5%	27,151	77.2%	22.8%	
Yes	2.5%	751	64.5%	35.6%	<0.001
Tuberculosis					
No	98.9%	27,622	77.1%	22.9%	
Yes	1.14%	280	53.6%	46.4%	<0.001
Malaria					
No	91.2%	25,755	77.9%	22.1%	
Yes	8.9%	2147	65.1%	34.9%	<0.001
Diarrhoea/gastroenteritis					
No	84.8%	24,080	77.7%	22.3%	
Yes	15.3%	3822	71.8%	28.2%	<0.001
Anemia					
No	95.5%	26,812	77.3%	22.7%	
Yes	4.5%	1090	67.3%	32.7%	<0.001

Notes: The weighted sample is used for this descriptive analysis. The *p*-values in the last column are obtained from chi-square tests comparing subjects in the underweight subsample with those in the not underweight subsample.

**Table 2:** Multivariate logistic regression results of factors associated with low BMI among older adults in India.

	(1)	(2)	(3)	(4)	(5)
Variables	Full Sample	Male Subsample	Female Subsample	Rural Subsample	Urban Subsample
	OR (95% CI)	OR (95% CI)	OR (95% CI)	OR (95% CI)	OR (95% CI)
Tobacco use (ref: No)					
Smoking	2.07 *** (1.79, 2.40)	1.93 *** (1.62, 2.31)	2.15 *** (1.65, 2.80)	2.17 *** (1.85, 2.55)	1.57 ** (1.07, 2.30)
Smokeless	1.26 *** (1.11, 1.42)	1.07 (0.89, 1.29)	1.46 *** (1.24, 1.73)	1.27 *** (1.11, 1.45)	1.15 (0.84, 1.57)
Both	1.74 *** (1.36, 2.22)	1.55 *** (1.18, 2.04)	4.26 *** (2.19, 8.28)	1.79 *** (1.38, 2.33)	1.55 (0.75, 3.17)
Food insecure	1.27 *** (1.10, 1.48)	1.39 *** (1.11, 1.75)	1.15 (0.95, 1.39)	1.26 *** (1.07, 1.48)	1.36 * (0.95, 1.95)
Controls:					
Age (ref: 60–69)					
70–79	1.19 *** (1.07, 1.33)	1.25 *** (1.08, 1.46)	1.14 (0.97, 1.33)	1.20 *** (1.06, 1.35)	1.25 * (0.96, 1.63)
80+	1.76 *** (1.44, 2.14)	1.84 *** (1.42, 2.38)	1.71 *** (1.28, 2.28)	1.63 *** (1.35, 1.97)	2.70 *** (1.53, 4.76)
Female	0.83 *** (0.74, 0.94)	-	-	0.86 ** (0.75, 0.98)	0.70 ** (0.52, 0.93)
Urban residence	0.50 *** (0.44, 0.58)	0.49 *** (0.41, 0.59)	0.53 *** (0.43, 0.65)	-	-
Not currently married	1.31 *** (1.18, 1.46)	1.27 *** (1.07, 1.50)	1.31 *** (1.13, 1.52)	1.32 *** (1.17, 1.48)	1.30 * (0.99, 1.70)
Region (ref: North)					
Central	1.74 *** (1.50, 2.02)	2.05 *** (1.65, 2.56)	1.49 *** (1.21, 1.83)	1.79 *** (1.52, 2.12)	1.54 ** (1.11, 2.15)
East	1.67 *** (1.44, 1.94)	1.55 *** (1.24, 1.94)	1.83 *** (1.50, 2.23)	1.78 *** (1.51, 2.09)	1.12 (0.78, 1.60)
Northeast	1.60 *** (1.33, 1.93)	1.43 ** (1.08, 1.90)	1.67 *** (1.29, 2.17)	1.74 *** (1.42, 2.12)	0.98 (0.54, 1.77)
West	1.30 *** (1.09, 1.56)	1.21 (0.93, 1.56)	1.39 ** (1.07, 1.79)	1.37 *** (1.11, 1.68)	1.02 (0.69, 1.51)
South	1.00 (0.84, 1.19)	0.98 (0.78, 1.23)	1.01 (0.77, 1.33)	1.02 (0.86, 1.22)	0.84 (0.53, 1.33)
Education (ref: No schooling)					
Middle or less	0.74 *** (0.66, 0.83)	0.78 *** (0.67, 0.90)	0.69 *** (0.56, 0.85)	0.76 *** (0.67, 0.87)	0.64 *** (0.48, 0.86)
At least secondary	0.45 *** (0.36, 0.57)	0.48 *** (0.37, 0.62)	0.26 *** (0.15, 0.45)	0.52 *** (0.39, 0.69)	0.31 *** (0.20, 0.46)
Not currently working	1.04 (0.93, 1.17)	1.04 (0.89, 1.21)	1.00 (0.85, 1.19)	1.01 (0.89, 1.14)	1.33 * (0.99, 1.78)
Caste (ref: ST)					
Scheduled Caste	0.85 * (0.71, 1.02)	0.856 (0.65, 1.11)	0.85 (0.66, 1.08)	0.84 * (0.69, 1.02)	0.83 (0.44, 1.59)
Other backward class	0.78 *** (0.66, 0.93)	0.74 ** (0.58, 0.95)	0.83 (0.65, 1.04)	0.78 *** (0.66, 0.93)	0.69 (0.37, 1.26)
None of above	0.61 *** (0.51, 0.74)	0.62 *** (0.47, 0.81)	0.61 *** (0.47, 0.78)	0.63 *** (0.52, 0.77)	0.49 ** (0.27, 0.91)
Religion (ref: Hindu)					
Muslim	0.88 (0.75, 1.03)	0.80 * (0.64, 1.00)	0.96 (0.78, 1.19)	0.87 (0.73, 1.04)	0.90 (0.65, 1.26)
Christian	0.81 (0.62, 1.07)	0.91 (0.59, 1.40)	0.74 (0.50, 1.07)	0.798 (0.59, 1.07)	0.98 (0.51, 1.90)
Others	0.96 (0.73, 1.26)	1.23 (0.85, 1.78)	0.72 (0.48, 1.08)	0.94 (0.70, 1.25)	1.09 (0.56, 2.11)
Wealth quintile (ref: Lowest)					
Second	0.91 (0.79, 1.05)	1.03 (0.84, 1.25)	0.82 * (0.68, 1.00)	0.94 (0.81, 1.09)	0.81 (0.58, 1.14)
Middle	0.80 *** (0.69, 0.93)	0.80 ** (0.64, 0.99)	0.82 * (0.67, 1.02)	0.83 ** (0.71, 0.97)	0.69 * (0.46, 1.03)
Fourth	0.61 *** (0.52, 0.71)	0.65 *** (0.52, 0.81)	0.57 *** (0.46, 0.71)	0.63 *** (0.53, 0.74)	0.58 ** (0.38, 0.88)
Highest	0.51 *** (0.43, 0.61)	0.52 *** (0.40, 0.66)	0.53 *** (0.42, 0.67)	0.52 *** (0.43, 0.63)	0.53 ** (0.32, 0.88)
Poor self-rated health	1.27 *** (1.12, 1.43)	1.23 ** (1.04, 1.47)	1.29 *** (1.09, 1.53)	1.24 *** (1.09, 1.41)	1.44 ** (1.03, 2.00)
1+ ADL	0.99 (0.87, 1.13)	0.99 (0.81, 1.22)	1.01 (0.85, 1.19)	1.05 (0.91, 1.21)	0.76 * (0.55, 1.04)
1+ IADL	1.14 ** (1.02, 1.28)	1.14 * (0.97, 1.33)	1.14 * (0.98, 1.34)	1.19 *** (1.06, 1.34)	0.88 (0.62, 1.25)
Chewing disability	1.29 *** (1.16, 1.42)	1.22 *** (1.06, 1.42)	1.33 *** (1.15, 1.53)	1.26 *** (1.13, 1.41)	1.44 *** (1.12, 1.84)
Hypertension	0.56 *** (0.49, 0.63)	0.53 *** (0.45, 0.63)	0.58 *** (0.49, 0.69)	0.57 *** (0.50, 0.64)	0.52 *** (0.38, 0.71)
Stroke	0.78 (0.56, 1.10)	0.79 (0.51, 1.24)	0.77 (0.48, 1.23)	0.87 (0.60, 1.25)	0.34 ** (0.13, 0.89)
Chronic heart diseases	0.66 ** (0.48, 0.91)	0.73 (0.47, 1.12)	0.59 ** (0.38, 0.90)	0.71 * (0.50, 1.02)	0.50 * (0.24, 1.02)
Cancer	0.95 (0.53, 1.70)	1.08 (0.43, 2.72)	0.80 (0.37, 1.72)	0.95 (0.46, 1.97)	1.02 (0.40, 2.58)
Chronic lung disease	1.69 *** (1.46, 1.97)	2.18 *** (1.79, 2.67)	1.25 ** (1.00, 1.56)	1.66 *** (1.40, 1.97)	1.83 *** (1.34, 2.52)
Diabetes	0.41 *** (0.34, 0.50)	0.52 *** (0.41, 0.67)	0.31 *** (0.23, 0.42)	0.44 *** (0.35, 0.55)	0.33 *** (0.23, 0.48)
Arthritis	0.69 *** (0.61, 0.79)	0.89 (0.74, 1.07)	0.55 *** (0.46, 0.66)	0.73 *** (0.63, 0.84)	0.52 *** (0.38, 0.71)
Jaundice/Hepatitis	1.30 * (0.99, 1.72)	1.26 (0.86, 1.82)	1.34 (0.90, 1.98)	1.19 (0.88, 1.61)	2.14 ** (1.08, 4.22)
Tuberculosis	2.17 *** (1.36, 3.47)	2.24 *** (1.24, 4.04)	2.38 *** (1.29, 4.40)	2.2 *** (1.29, 3.74)	2.12 * (0.97, 4.63)
Malaria	1.19 ** (1.02, 1.40)	1.36 ** (1.08, 1.73)	1.05 (0.85, 1.29)	1.20 ** (1.01, 1.42)	1.28 (0.81, 2.03)
Diarrhoea/gastroenteritis	0.93 (0.81, 1.07)	0.80 ** (0.66, 0.98)	1.06 (0.88, 1.27)	0.94 (0.81, 1.09)	0.94 (0.67, 1.32)
Anemia	1.47 *** (1.19, 1.81)	1.77 *** (1.30, 2.40)	1.35 ** (1.01, 1.80)	1.53 *** (1.22, 1.92)	1.08 (0.62, 1.86)
Pseudo R^2^	0.148	0.157	0.153	0.105	0.178
Observations	27,902	13,487	14,415	18,715	9187

Notes: * *p* < 0.01, ** *p* < 0.005, *** *p* < 0.001. Individual-level weights are applied.

**Table 3:** Multivariate logistic regression results with interaction terms for tobacco use.

	Model 1	Model 2
Variables	OR (95% CI)	OR (95% CI)
Tobacco use (ref: No)		
Smoking	1.86 *** (1.55, 2.22)	1.62 *** (1.25, 2.11)
Smokeless	1.28 *** (1.11, 1.478)	1.09 (0.87, 1.37)
Both	1.69 *** (1.23, 2.32)	1.09 (0.70, 1.68)
Education (ref: No schooling)		
Middle or less	0.70 *** (0.59, 0.82)	0.74 *** (0.66, 0.83)
At least secondary	0.46 *** (0.34, 0.62)	0.45 *** (0.36, 0.57)
Wealth quintile (ref: Lowest)		
Second quintile	0.92 (0.80, 1.05)	0.83 * (0.68, 1.00)
Middle quintile	0.80 *** (0.69, 0.94)	0.76 ** (0.62, 0.94)
Fourth quintile	0.61 *** (0.52, 0.71)	0.48 *** (0.39, 0.60)
Highest quintile	0.52 *** (0.43, 0.62)	0.46 *** (0.37, 0.58)
Interaction terms for tobacco use:		
Tobacco use and Education (ref: No tobacco#no schooling)		-
Smoking tobacco#Middle or less	1.33 * (0.99, 1.79)	
Smoking tobacco#At least secondary	1.27 (0.74, 2.15)	
Smokeless tobacco#Middle or less	1.02 (0.78, 1.33)	
Smokeless tobacco#At least secondary	0.76 (0.47, 1.22)	
Both tobacco #Middle or less	1.13 (0.69, 1.85)	
Both tobacco #At least secondary	0.88 (0.36, 2.19)	
Tobacco use and Wealth (ref: No tobacco#lowest quintile)	-	
Smoking tobacco#second quintile		1.37 (0.94, 2.00)
Smoking tobacco#middle quintile		1.12 (0.75, 1.66)
Smoking tobacco#fourth quintile		1.70 ** (1.12, 2.57)
Smoking tobacco#highest quintile		1.47 * (0.95, 2.28)
Smokeless tobacco#second quintile		1.20 (0.87, 1.64)
Smokeless tobacco#middle quintile		1.09 (0.77, 1.53)
Smokeless tobacco#fourth quintile		1.55 ** (1.08, 2.22)
Smokeless tobacco#highest quintile		1.13 (0.73, 1.75)
Both tobacco#second quintile		1.52 (0.82, 2.80)
Both tobacco#middle quintile		1.69 (0.89, 3.18)
Both tobacco#fourth quintile		2.97 *** (1.55, 5.70)
Both tobacco#highest quintile		1.71 (0.66, 4.42)
Observations	27,902	27,902

Notes: * *p* < 0.01, ** *p* < 0.005, *** *p* < 0.001. # is used to denote an interaction between pairs of variables in the table. Individual-level weights are applied. These regression results are adjusted for all other covariates as listed in Table 2 but omitted here for brevity.

## Data Availability

The data are publicly available for download to registered users on the LASI website (https://lasi-india.org/ (accessed on 11 August 2024)).
